# RepA-WH1, the agent of an amyloid proteinopathy in bacteria, builds oligomeric pores through lipid vesicles

**DOI:** 10.1038/srep23144

**Published:** 2016-03-17

**Authors:** Cristina Fernández, Rafael Núñez-Ramírez, Mercedes Jiménez, Germán Rivas, Rafael Giraldo

**Affiliations:** 1 Department of Cellular and Molecular Biology Centro de Investigaciones Biológicas-CSIC, E28040 Madrid, Spain; 2 Electron Microscopy Facility, Centro de Investigaciones Biológicas–CSIC, E28040 Madrid, Spain

## Abstract

RepA-WH1 is a disease-unrelated protein that recapitulates in bacteria key aspects of human amyloid proteinopathies: i) It undergoes ligand-promoted amyloidogenesis *in vitro*; ii) its aggregates are able to seed/template amyloidosis on soluble protein molecules; iii) its conformation is modulated by Hsp70 chaperones *in vivo*, generating transmissible amyloid strains; and iv) causes proliferative senescence. Membrane disruption by amyloidogenic oligomers has been found for most proteins causing human neurodegenerative diseases. Here we report that, as for PrP prion and α-synuclein, acidic phospholipids also promote RepA-WH1 amyloidogenesis *in vitro*. RepA-WH1 molecules bind to liposomes, where the protein assembles oligomeric membrane pores. Fluorescent tracer molecules entrapped in the lumen of the vesicles leak through these pores and RepA-WH1 can then form large aggregates on the surface of the vesicles without inducing their lysis. These findings prove that it is feasible to generate *in vitro* a synthetic proteinopathy with a minimal set of cytomimetic components and support the view that cell membranes are primary targets in protein amyloidoses.

A long-standing issue on human amyloidoses is the identity of the primary cellular target for the protein aggregates that leads to disease^1^,^2^,^3^. A number of different, often interwoven, pathways have been described as involved in the etiology of most neurodegenerative proteinopathies, including gain of toxicity phenotypes such as mitochondrial damage with generation of reactive oxygen species^4^,^5^, or the co-aggregation and sequestering of essential cell factors^6^,^7^. However, direct damage to the cell membrane^8^ is the mechanism most commonly found when amyloidoses are addressed *in vitro*, i.e. in studies on the interaction between amyloid aggregates or fibres with reconstituted cytomimetic lipid vesicles (liposomes), such as large or giant unilamellar vesicles (LUVs and GUVs, respectively)^9^,^10^. These findings are compatible with others gathered from the incubation of amyloid aggregates with cultured cells, thus assessing their toxicity^11^,^12^,^13^,^14^. Nowadays it is accepted that membrane targeting is an early route towards amyloid cytotoxicity and cell death.

Bacteria are prokaryotic microorganisms naturally freed of amyloid ‘diseases’, although they use extracellular, secreted amyloids as functional devices to scaffold biofilms or to regulate the activity of antibiotic peptides^15^,^16^. Within a Synthetic Biology framework, we have recently undertaken the bottom-up design of a model amyloid proteinopathy in the bacterium *Escherichia coli*^17^. In a first stage, we engineered RepA, a protein coded by plasmid pPS10, by deleting its C-terminal domain (WH2) thus leaving the N-terminal *winged-helix* domain (WH1)^18^. This decoupled DNA-promoted conformational transitions in RepA-WH1 from its natural role: enabling RepA as a DNA replication initiator^19^. After incorporating in RepA-WH1 a mutation (A31V) known to enhance protein-protein interactions expanding the DNA replication capabilities of pPS10^20^,^21^, a DNA-modulated amyloidogenic part was generated and tested successfully *in vitro*^22^,^23^. RepA-WH1 thus shares with the mammalian prion PrP the ability of nucleic acids ligands to promote amyloidogenesis^24^. In a second step, RepA-WH1 was fused at its C-terminus to a fluorescent protein (mCherry), thus replacing the original WH2 domain in RepA. When expressed in *E. coli*, this protein fusion first assembles amyloid precursors at the nucleoid, which then are excluded to the cytoplasm^25^. RepA-WH1 aggregates elicit a ‘proteinopathy’, as became evident in time-lapse microscopy studies showing a decrease in the proliferative fitness (i.e., a significant increase in the generation time) of the cells^26^,^27^, a phenotype linked to ‘aging’ in bacteria^28^. Cytoplasmic RepA-WH1 aggregates were able to template their amyloid conformation on soluble protein molecules both *in vitro*^26^ and *in vivo*^29^, a hallmark of the molecular transmissibility of prions^30^,^31^. Although the RepA-WH1 aggregates are ‘vertically’ inherited from mother to daughter cells, they lack ‘horizontal’ microbiological infectivity, thus they can be regarded as a prionoid^32^, which together with its lack of significant sequence similarity to any mammalian protein qualify RepA-WH1 as biosafe. Subsequent microfluidic studies *in vivo* revealed that RepA-WH1 propagates as two distinct conformational variants (or ‘strains’) showing different aggregation morphologies and degrees of toxicity, which are epigenetically inherited by the bacterial offspring along generations^28^. DnaK, the Hsp70 chaperone in *E. coli*, was identified as the single cell factor modulating the switch to the variant with a lower toxicity and enabling the generation of transmissible particles^27^. Overall, the RepA-WH1 prionoid matches many of the features of clinically relevant amyloid proteinopathies, providing a simple molecular platform to address in bacteria the complexity of protein amyloidosis.

In this work we have explored the interaction between RepA-WH1 and vesicles of different sizes (LUVs/GUVs) and phospholipid (PL) composition. Incubation with vesicles either resembling *E*. c*oli* cell membrane or including PLs with acidic polar heads (aPLs: phosphatidyl glycerol, cardiolipin) promoted the assembly of RepA-WH1 as pre-amyloid oligomers and fibrils. Furthermore, RepA-WH1 binding to LUVs and GUVs released a fluorescent tracer (calcein) pre-confined in the vesicles, thus enabling to follow the kinetics of membrane leakage by means of fluorescence spectroscopy (LUVs) or microscopy (GUVs). RepA-WH1 dimers were more efficient in targeting membranes than preformed aggregates of the protein. Membrane disruption in GUVs did not result in lysis of the vesicles, suggesting the assembly of discrete oligomeric protein pores by RepA-WH1, which were visualized by transmission electron microscopy (TEM). These *in vitro* assays allowed testing a number of natural polyphenolic compounds known to counteract the amyloidosis of proteins involved in human disease. In common with the mammalian prion PrP^24^,^33^,^34^, having both nucleic acids and aPLs as effectors of amyloidosis qualifies the prionoid RepA-WH1 as a robust proxy to model human amyloid proteinopathies through minimalist approaches, either in bacteria *in vivo*^26^,^27^ or, as reported here, in cytomimetic assays *in vitro*.

## Results

### Binding to the internal membrane or acidic phospholipid vesicles promotes RepA-WH1 aggregation

**Figure 1:**
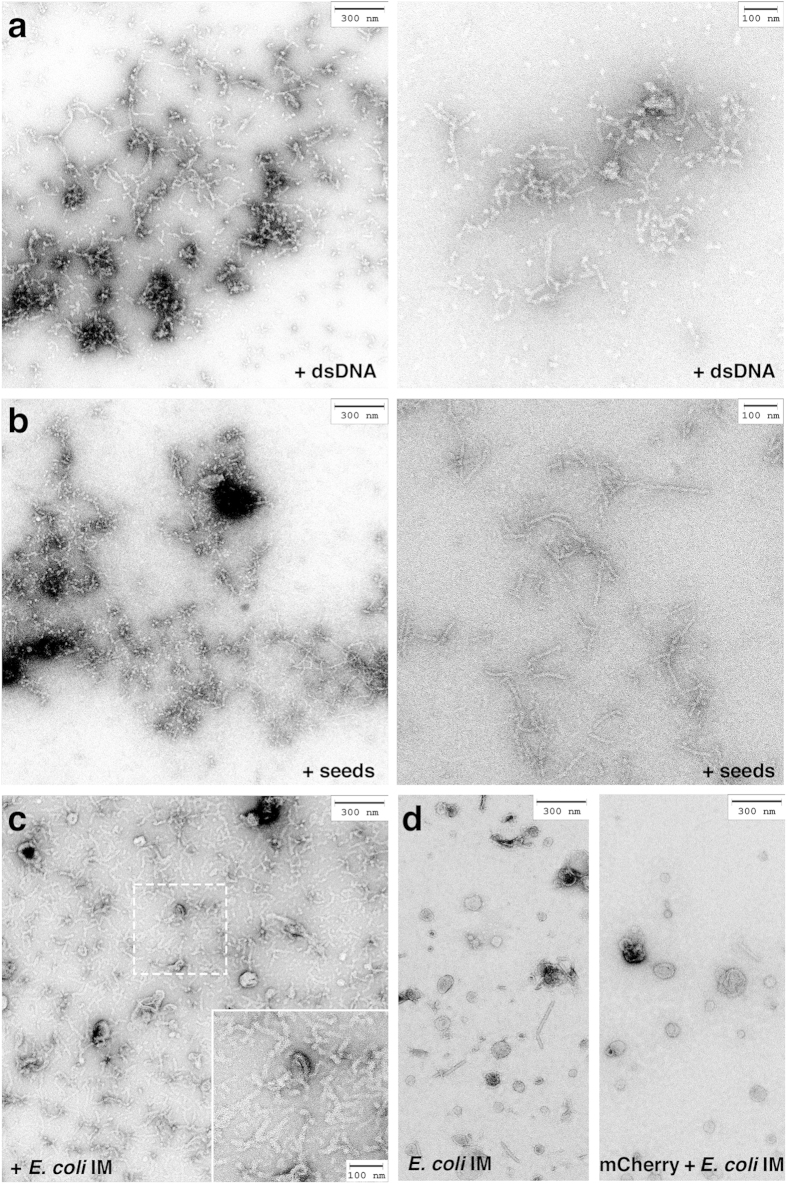
RepA-WH1(A31V)-mCherry aggregates in the presence of DNA, *ex-vivo* purified amyloid seeds, and the internal membrane of *E. coli*. Negatively stained EM images of RepA-WH1(A31V)-mCherry fibres assembled *in vitro* in the presence of: (**a**). Effector dsDNA (20 days, 4 °C). (**b**) Aggregate seeds (same conditions as in a). (**c**) *E. coli* internal membrane fraction (2 h, room temperature). Inset: magnification of the boxed sector. (**d**) Controls: *E. coli* internal membrane alone (*left* panel) and upon incubation with mCherry (*right*).

Previously published results indicate that binding of the bacterial RepA-WH1 prionoid, especially of its point mutant A31V, to different dsDNA sequences leads to the assembly of the protein into distinct amyloid nanostructures, including either oligomers, spherical aggregates of various sizes, or well ordered fibres^22^,^23^. Inspired by the fact that, together with RNA, phospholipids (PLs) are key cofactors for PrP replication *in vitro*^33^,^34^, we decided to explore if PLs could also have a role in RepA-WH1 amyloidogenesis. We studied the interaction between the hyper-amyloidogenic variant RepA-WH1(A31V) fused to the fluorescent reporter mCherry, because this chimeric protein had been defined as the most cytotoxic *in vivo*, and the internal membrane fraction of *E. coli*, since this is the host where the RepA-WH1 proteinopathy had been characterized^26^,^27^. In these *in vitro* assays (Fig. 1), membrane preparations surpassed the most efficient effector DNA sequence in getting visible aggregation at the electron microscope, i.e. from ≥20 days (*opsp* dsDNA)^22^ to barely 2 h (membrane), and relieved the need for a crowding agent in the reaction (see Methods). However, instead of highly ordered, long and thick straight amyloid fibres as for RepA-WH1(A31V)^22^,^35^, shorter, curved and thinner protofibrils were obtained for the protein fused to mCherry. As the same inner membrane preparations were efficient in promoting the assembly of the standard mature RepA-WH1(A31V) multi-filament fibres (Supplementary Fig. 1)^22^,^35^, the most likely explanation is that the C-terminally fused mCherry protein would be imposing steric constrains to the lateral assembly of the protofibrils into the mature fibres. In parallel, as controls, we studied the association states (Supplementary Fig. 2a) and the secondary structures (Supplementary Fig. 2b,c) of RepA-WH1(A31V)-mCherry, either in the presence or in the absence of a His_6_ N-terminal tag, and of isolated His_6_-mCherry. The latter was monomeric and its hexa-histidine tag did not alter the association state or the structure of the protein. Although the unfused RepA-WH1(A31V) was dimeric^22^,^35^, RepA-WH1(A31V)-mCherry included, besides dimers, a significant aggregated fraction as shown by the dispersion of the sedimentation coefficients (s) towards higher values, attributable to the presence of oligomers (Supplementary Fig. 2a). CD spectroscopy revealed that the fusions were thermally stable (Tm values ≈90 °C), albeit not matching the extreme stability of their parental RepA-WH1(A31V)^22^ (Supplementary Fig. 2b), suggesting some destabilization of this domain by the C-terminal mCherry. The spectra of the individual components in the fusion were additive, i.e., their algebraic addition nearly matched the spectrum of the whole protein (Supplementary Fig. 2c), indicating that RepA-WH1 and mCherry were essentially independent folding modules.

### RepA-WH1 aggregation on the surface of model lipid vesicles leads to membrane leakage

**Figure 2:**
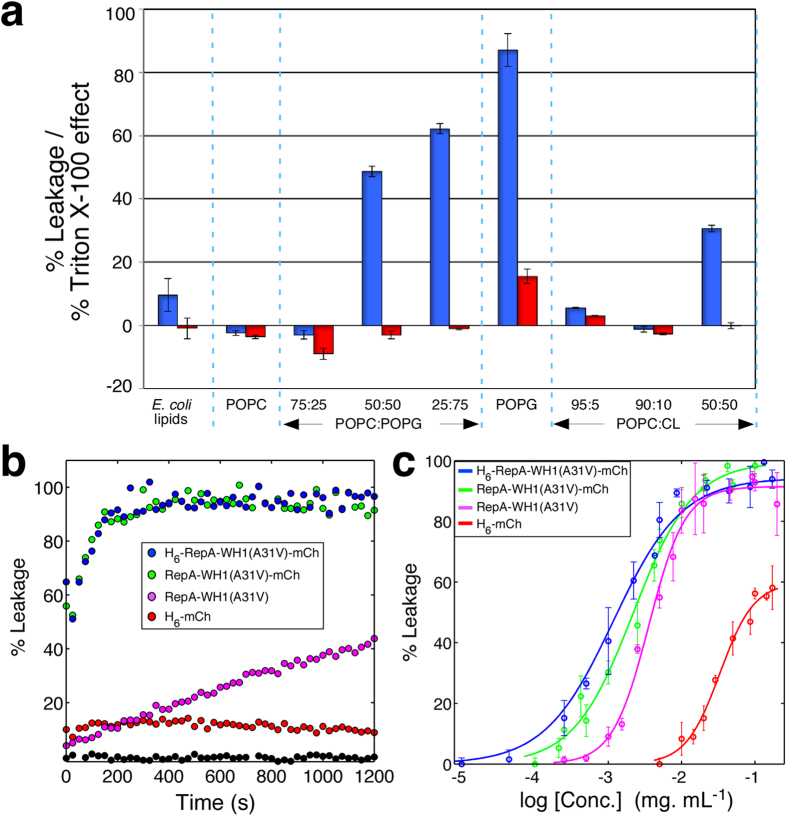
RepA-WH1 induces permeation of liposome vesicles in suspension. (**a**) Calcein efflux from LUVs (30 μM lipid mixtures) was induced by 0.05 μM of RepA-WH1(A31V)-mCherry. Histograms show the average of three independent experiments. (**b**) Kinetics of protein-induced membrane permeabilization. Proteins (0.5 μM) were added (t = 0) to calcein-filled POPC:POPG (1:1) LUVs (30 μM). Leakage after complete disruption of all vesicles by Triton X-100 was set to 100%. Background fluorescence is also plotted (black). (**c**) Dependence of the leakage from POPC:POPG LUVs on the concentration of proteins. Solid lines correspond to the best fit to an empirical Hill equation.

A commonly proposed mechanism for amyloid cytotoxicity implies binding and leakage of cell membranes^8^. The interaction of RepA-WH1 with suspensions of large unilamellar vesicles (LUVs; ≈100 nm ∅) with various lipid compositions was assayed (Fig. 2). LUVs are appropriate probes for getting kinetic insight into membrane targeting by amyloids because, due to their small radii, they exhibit reduced light scattering and have a large surface curvature, thus being sensitive to protein insertion into the lipid bilayer^8^. In these assays, LUVs bore the fluorophore calcein encapsulated: fluorescence emission increases if calcein is released into the medium, thus acting as a probe for membrane leakage. The lipid composition that led to maximum fluorescence emission upon incubation with RepA-WH1 included mixtures, with an optimum around 50%, of the zwitterionic phospholipid POPC (in short PC) and acidic phospholipids (aPLs, with negatively charged polar heads), such as POPG (PG) or cardiolipin (CL). Higher fractions of the negatively charged PLs resulted in intrinsic destabilization of the vesicles, leading to some leakage induced even by mCherry (Fig. 2a). Time-course measurements of LUVs leakage (Fig. 2b,c) showed that RepA-WH1(A31V)-mCherry, either with or without the His_6_ tag, damaged the vesicles readily, exhibiting a logarithmic release profile, whereas a RepA-WH1(A31V) control without the mCherry moiety showed a nearly linear plot, and mCherry barely had any fluorescence releasing activity by itself. The observed differences also suggest that the His_6_-tag enhances binding affinity of RepA-WH1(A31V)-mCherry to lipids: at the mild acidic pH of the experiments (6.0), the protonated imidazoles of the six histidines should increase the electrostatic attraction of the protein for aPLs. However, the His_6_-tag had not a major role in vesicle leakage because, as noted above, His_6_-mCherry was inert in the calcein release assay.

In the absence of the C-terminal fusion to mCherry, RepA-WH1(A31V) had its capacity to leak phospholipid vesicles reduced (Fig. 2b,c). This could be attributed to a higher rate of amyloidogenesis for the chimera with the fluorescent protein reporter, as suggested by its lower stability (Supplementary Fig. 2b). Alternatively, since the fusion to mCherry led to a net increase in the fraction of oligomerised RepA-WH1(A31V), especially for the His6-tagged variant (Supplementary Fig. 2a), oligomers might have higher activity on membranes than dimers. To address this possibility, the dimeric and oligomeric components in RepA-WH1(A31V)-mCherry preparations were fractionated by gel filtration and analysed through in-line multi-angle light scattering (SEC-MALS) (Supplementary Fig. 3a). Both fractions were assayed for their ability to leak calcein encapsulated in LUVs made of 1:1 mixtures of PC and PG (Supplementary Fig. 3b), as indicated above. Interestingly, when assayed at equal concentrations, the dimeric protein fraction was twice as efficient as the oligomeric one in promoting calcein release. This finding suggests that binding of dimeric RepA-WH1 to the membrane must be directly linked to the event of vesicle leakage, whereas the protein aggregates generated in the absence of effector lipids are less competent to bind and/or leak membranes.

**Figure 3:**
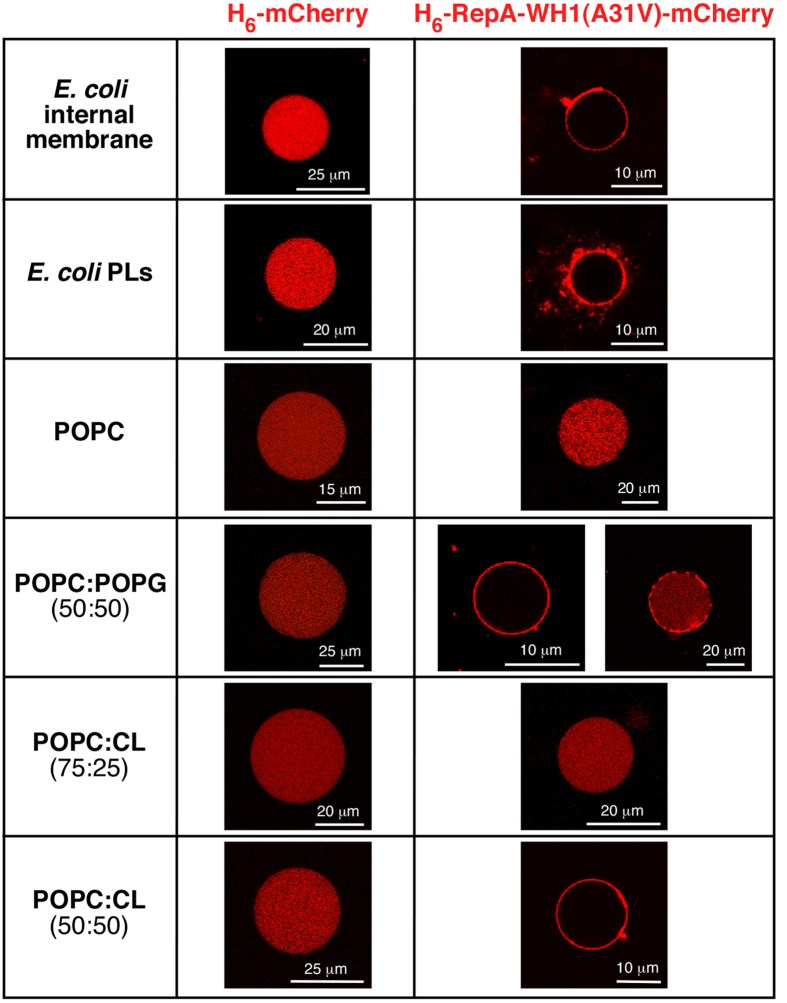
RepA-WH1(A31V)-mCherry binds to and aggregates on GUVs. Single equatorial confocal sections of liposomes formed from hybrid films of agarose and lipids. Lipid compositions used: purified from *E. coli* inner membrane; *E. coli* total lipid extract (67.0% PE, 23.2 & PG, 9.8% CL); POPC (1-palmitoyl–2-oleoyl-*sn*-glycero-3-phosphocholine); 1:1 mixture of POPC and POPG (1-palmitoyl-2-oleoyl-*sn*-glycero-3-[phospho-(1′-*rac*-glycerol)]); 3:1 mixture of POPC and cardiolipin (CL; 1,3-*bis*(*sn*-3′-phosphatidyl)-*sn*-glycerol); 1:1 mixture of POPC and CL. Lipids were incubated at 37 °C during GUV formation with either H_6_-RepA-WH1(A31V)-mCherry or H_6_-mCherry (4 μM).

To get further insight into the interaction between RepA-WH1 and the membranes, we monitored the interaction between the prionoid and giant unilamellar vesicles (GUVs; 5–50 μm Ø), made of different lipid compositions and using diverse assembly procedures. Vesicles were visualized by means of confocal laser fluorescence microscopy (Fig. 3). In comparison with the control (purified mCherry), which remained as diffused fluorescence (i.e., soluble protein) in the lumen of the vesicles, RepA-WH1(A31V)-mCherry became visible as protein aggregates on the surface of the GUVs when the vesicles were prepared with purified bacterial internal membranes. This observation was repeated with GUVs made of PLs with the average composition characteristic of *E. coli* inner membrane. Aggregation at the surface of the vesicles was also evident with less complex PLs compositions, i.e., if GUVs included aPLs (PG or CL). It is noteworthy that concentrations of aPLs >50% could not be reproducibly tested, due to their destabilizing effect on GUVs. However, RepA-WH1(A31V)-mCherry remained soluble, as the mCherry control did, if the lipids were exclusively neutral (PC). The inference from the LUVs and GUVs minimal model membranes is that aPLs present in the *E. coli* internal membrane act as co-factors promoting RepA-WH1 amyloidogenesis.

**Figure 4:**
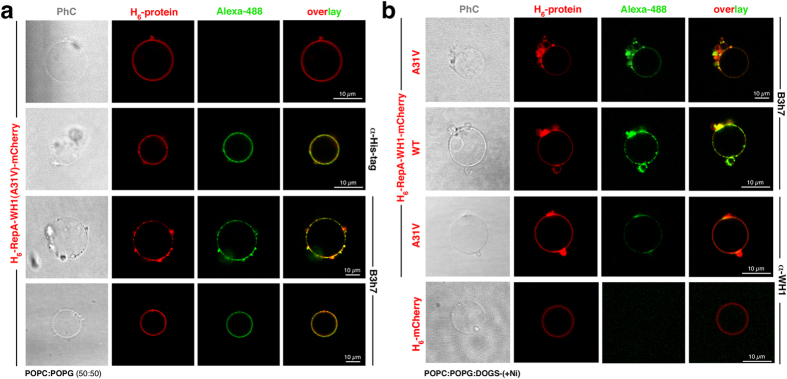
RepA-WH1(A31V)-mCherry forms amyloidogenic aggregates on GUVs. (**a**) Single equatorial confocal sections of POPC:POPG (1:1) GUVs incubated with H_6_-RepA-WH1(A31V)-mCherry (red) and antibodies (green): B3h7, specific for pre-amyloid oligomers of RepA-WH1, or anti-His. PhC: phase contrast imaging. (**b**) Higher aggregation of His_6_-RepA-WH1(A31V)-mCherry (red) on GUVs can be achieved by including in the vesicles a metal-chelating lipid (DOGS-NTA; 1,2-di-(9Z-octadecenoyl)-*sn*-glycero-3-[(N-(5-amino-1-carboxypentyl)iminodiacetic acid)succinyl]). Binding to lipids through metal coordination forced aggregation of the mildly amyloidogenic His_6_-RepA-WH1(WT)-mCherry variant, otherwise soluble. His_6_-mCherry, albeit also targeted to membranes, did not aggregate. B3h7 and α-WH1 antibody labels (green).

In order to inquire if the lipid-promoted RepA-WH1(A31V) aggregates had amyloidogenic nature, we probed the protein-bound GUVs by means of immunocytochemistry (Fig. 4) using B3h7, an antibody specific for RepA-WH1 oligomers on-pathway towards the assembly of amyloid fibres^25^. B3h7 bound to the aggregates made by the hyper-amyloidogenic protein RepA-WH1(A31V)-mCherry (Fig. 4a). This antibody also recognized the aggregates assembled by RepA-WH1(WT)-mCherry, an otherwise soluble, mildly amyloidogenic variant of the prionoid^26^,^27^,^29^, when this protein was clustered (and thus forced to aggregate) on the membrane by coordinating its N-terminal His_6_ tag with the Ni^2+^-activated polar head of a chelating lipid (DOGS-NTA) (Fig. 4b). On the contrary, mCherry was not bound either by the B3h7 antibody or by α-WH1, a conformation-unspecific anti-RepA-WH1 polyclonal antibody^25^ (Fig. 4b). Overall, the results shown in this section are compatible with enhanced pro-amyloidogenic aggregation of RepA-WH1 upon binding to lipid vesicles.

**Figure 5:**
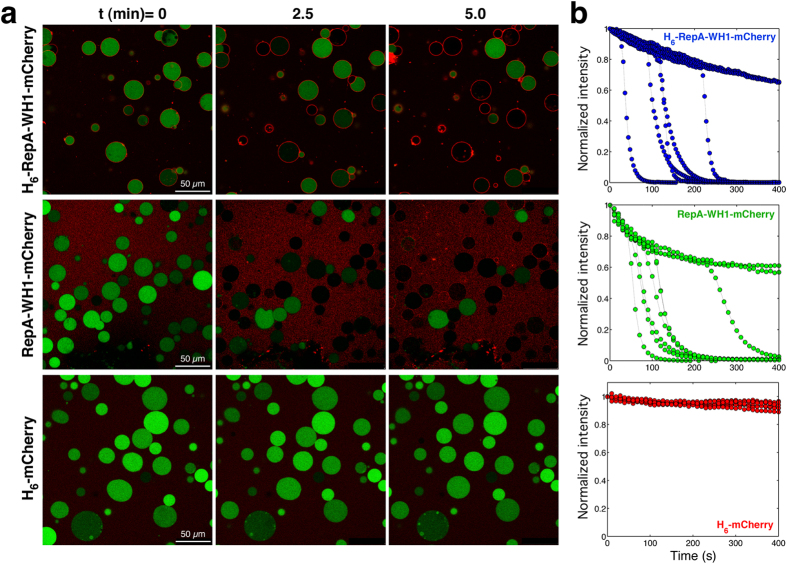
Single vesicle assessment of RepA-WH1 promoted permeation of model membranes. (**a**) Confocal microscopy images of POPC:POPG (1:1) GUVs filled with calcein (green). Time-elapsed images follow calcein efflux, i.e. progressive decrease in fluorescence intensity, from every GUV upon addition of (His_6_-tagged or untagged) RepA-WH1(A31V)-mCherry (0.4 μM). See Movies S1-S3. 150 vesicles of each kind from several experiments were counted and the % of leaked GUVs determined: H6-RepA-WH1(A31V)-mCherry, 69.5 +/−9.7; RepA-WH1-mCherry, 73.4 +/−9.1; H_6_-mCherry, 14.0 +/−4.5. (**b**) Calcein fluorescence intensity *vs*. time plotted for 8 individual vesicles in a. Fluorescence was normalized to the area of the confocal section of vesicles at t = 0. Exponential fluorescence decay indicates leakage from a GUV, whereas lower slope curves are associated with photobleaching (e.g., in GUVs incubated with mCherry). Persistence of the diameters of vesicles (see also Fig. 3) suggests that leakage by RepA-WH1 occurs with no severe disruption of the lipid bilayer, as expected upon the assembly of protein pores.

To achieve single vesicle resolution in the leakage assays, we incubated the different protein constructs with GUVs in which calcein had been encapsulated (Fig. 5a). In these assays, fluorescence emission decreases upon release of calcein into the medium. The result was that RepA-WH1(A31V)-mCherry, whether it included the His_6_ tag (Supplementary Movie 1) or not (Supplementary Movie 2), led to vesicle leakage, whereas the mCherry control did not (Supplementary Movie 3). Monitoring fluorescence intensity decay of the green channel for several single vesicles in such time-elapsed movies defined single exponential kinetics for RepA-WH1 elicited calcein efflux from the vesicles (Fig. 5a). Calcein leaked almost completely in one stage at a concentration of protein of 0.4 μM (K_leak_ = 0.071 ± 0.033 s^−1^). The curves from both His_6_-tagged and untagged RepA-WH1(A31V)-mCherry were clearly distinguishable from fluorescence photobleaching, which in control intact vesicles, as in the case of incubation with mCherry, resulted in plots with much lower slopes. It is noteworthy that the experiments carried out with GUVs (see also Figs 3 and 4) provided no evidence on a massive lytic disruption of the vesicles upon RepA-WH1 binding.

### Inhibitors of amyloidogenesis neutralize membrane damage by the RepA-WH1 prionoid

**Figure 6:**
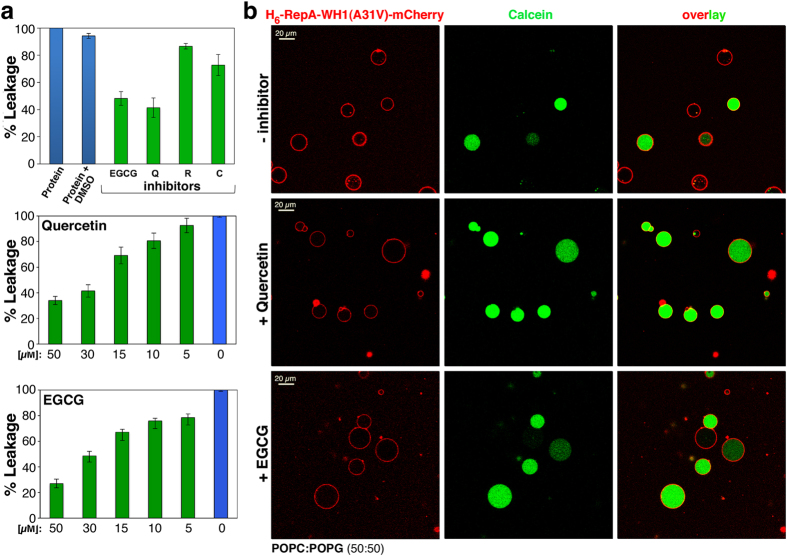
Natural poly-phenolic inhibitors of amyloidogenesis interfere with calcein release from lipid vesicles promoted by RepA-WH1(A31V)-mCherry. (**a**) LUVs: Epigallocathecin-3-gallate (EGCG), quercetin (Q), resveratrol (R) and curcumin (C) were tested (50 μM) as in Fig. 2. Data from three independent experiments were normalized to the fluorescence released by incubation with protein alone. Solvent (DMSO) had no significant effect on vesicle leakage. Q and EGCG were the most efficient inhibitory molecules (60-40%) and thus they were explored further by serial dilution (*middle* and *bottom* panels). (**b**) GUVs: The two polyphenols more efficient in (a) were also assayed in giant vesicles. Both Q (*middle* row) and EGCG (*bottom*) (50 μM) significantly reduced protein-induced damage of GUVs (see Fig. 5). For a quantitative assessment, % of leaked GUVs was accounted from three independent experiments (≈200 vesicles each kind): control, 55.3 +/−8.7; EGCG, 17.3 +/−2.5; Q, 8.0 +/−5.6.

Lipid vesicles have been recently used to monitor the interference of small molecules, such as natural polyphenols^36^, with the amyloidogenesis of α-synuclein on membranes^37^. We thus tested the possible interference of the polyphenols epigallocathecin-3-gallate (EGCG), quercetin (Q), resveratrol (R) and curcumin (C) on vesicle leakage by assembled RepA-WH1 pores. All these compounds exhibited some inhibitory effect on calcein release from LUVs, as elicited by RepA-WH1(A31V)-mCherry, ranging from ≈10% (R) to ≈60% (Q) (Fig. 6a). Therefore, the two compounds with best inhibitory properties (Q and EGCG) were explored in more detail. In the interval between 5–60 μM, both Q and EGCG showed a linear response with concentration. The action of polyphenols on the leakage promoted by RepA-WH1 in the GUVs membrane model was also tested (Fig. 6b): both Q and EGCG substantially decreased the fraction of vesicles that released calcein upon incubation with the prionoid, confirming the protective role of polyphenols on protein-promoted damage to membranes.

Since we had found a preference for soluble RepA-WH1(A31V)-mCherry dimers over preassembled oligomers in binding and leaking lipid vesicles (Supplementary Fig. 3a,b), we separately tested in the calcein release assay using LUVs the most active polyphenol (Q) on both the dimeric and oligomeric fractions of the protein. The result (Supplementary Fig. 3c) showed that quercetin reduced the release of the fluorophore from the vesicles by both dimers and oligomers of RepA-WH1(A31V)-mCherry, although more efficiently (up to twice, at 10 μM Q) for the latter than for the former. This suggests that polyphenols most probably both interfere with a process involved in membrane leakage, i.e. a lipid-promoted structural transformation of dimers, and remodel protein aggregates yielding species less active towards membranes.

### Assembly of oligomeric pores as the mechanism for membrane damage by RepA-WH1

**Figure 7:**
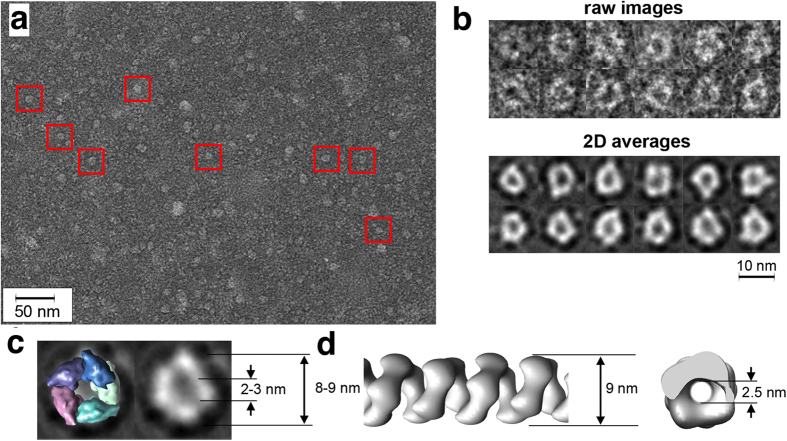
Electron microscopy of H6-RepA-WH1(A31V)-mCherry oligomers over *E. coli* lipids monolayers. (**a**) General view of the negatively stained protein oligomeric rings. Some representative particles are boxed. (**b**) Galleries showing selected raw images (*top*) and reference-free 2D average projections (*bottom*) of the oligomeric rings. (**c**) Five RepA-WH1 molecules (rainbow coloured), head-to-tail arranged in their monomeric conformation^19^, manually fitted to the mean projection of a pore. The external and internal dimensions of the channel are indicated (*right*). (**d**) The average dimensions of the pores are similar to those measured for the section of the helical, tubular fibrils assembled by RepA-WH1, albeit in the latter the monomers accommodate a significant structural distortion^35^.

The results obtained with both LUVs and GUVs converged towards an scenario in which RepA-WH1(A31V)-mCherry molecules upon binding to lipid vesicles cause their leakage, with a preference for aPLs as promoters of protein amyloidogenesis. Regarding the mechanism for such damage to the membrane, one possibility is the formation of protein pores because there was no lytic disruption of the vesicles, or even any alteration in their appearance, as previously found for α-synuclein^38^. However, an alternative lipid-extraction mechanism could not be excluded^8^. Therefore, we aimed to directly visualize the complexes assembled by RepA-WH1 at a model lipid membrane. After probing a number of different conditions, we succeeded in assembling oligomeric RepA-WH1(A31V)-mCherry particles in monolayers made of PLs with the average composition found in *E. coli*, which were pre-casted on carbon-coated EM grids (Fig. 7a). Particles were negatively stained with a uranyl salt and EM images were acquired under minimal radiation doses. RepA-WH1 particles were individually picked, classified and 2D-projections reconstructed following standard procedures (see Methods) (Fig. 7b). Protein oligomeric rings were readily visualised, albeit their shapes were heterogeneous. Such heterogeneity, which suggests a dynamic nature for the pores, precluded the assignment of a unique number of subunits in their composition. The overall dimensions of the protein pores were 8–9 nm for the exterior of the rings and 2–3 nm for the internal channels (Fig. 7c). The average 2D-projections are fully compatible with head-to-tail arranged RepA-WH1 monomers, which should proceed from dissociation of protein dimers upon binding to the effector lipids, as reported for RepA-dsDNA-promoted RepA-WH1 amyloidogenesis^38^, as building blocks of the channels. These results directly support that the mechanism responsible for vesicle leakage promoted by RepA-WH1, and most probably for triggering the toxicity of the prionoid in bacterial cells, is the formation of pores at the membrane.

## Discussion

The results reported here point to a possible primary target for the cytotoxicity expressed by the RepA-WH1 prionoid in *E. coli*: the bacterial internal cell membrane. Three distinct mechanisms have been proposed for membrane disruption by amyloidogenic proteins^8^, including: i) the formation of pores by the assembly of protein monomers into ring-shaped oligomers; ii) membrane thinning and iii) lipid (detergent-like) extraction. Since the last mechanism should imply the lysis of a significant fraction of the vesicles, which was not the case under our experimental conditions, and the second one seems incompatible with the observed persistence of aggregates bound to the membranes (Figs 3, 4, 5), the formation of pores by protein insertion into the lipid bilayer, as first proposed for Aβ and α-synuclein^39^, was the most likely mechanism for vesicle leakage induced by RepA-WH1.

We have shown here that RepA-WH1 dimers, rather than preassembled oligomers, are the most active species in inducing leakage through bilayers including acidic phospholipids (Supplementary Fig. 3a,b). Furthermore, through *E. coli* lipids coating EM grids, RepA-WH1 is able to assemble as channel pores whose average dimensions match those for the pores built by other amyloidogenic proteins^8^. Although the number of RepA-WH1 subunits constituting the walls of the pores is variable (with a mode of five) their densities are clearly compatible with protein monomers arranged head-to-tail, rather than with dimers that are disposed head-to-head and related by two-fold symmetry (Fig. 7c)^19^. This is relevant because, as for dsDNA and seed-promoted amyloidogenesis^35^, binding of RepA-WH1 to membranes consequently implies dimer dissociation and pore assembly through a monomeric intermediate. For distinct proteins and under different conditions, the conversion of membrane-bound and ring-shaped oligomeric pores into fibrils has been proposed as a plausible further step in amyloidogenesis^40^,^41^,^42^,^14^. Interestingly, in the presence of effector dsDNA molecules or amyloid seeds, RepA-WH1 monomers assemble *in vitro* as hollow single or double helical nanotubes with an internal diameter ≈2.5 nm^35^. This is well within the range measured here for the RepA-WH1 channels *in vitro* (≈2.0–3.0 nm) (Fig. 7d), making feasible that the bacterial prionoid could also build ring-shaped pores upon insertion into the membrane *in vivo*. Therefore, this could also be the case for RepA-WH1 once assembled into the membrane: the dynamic nature of the pores would allow for ring opening and the subsequent helical extrusion of a free protein end, enabling seeding and growth of an amyloid protofilament out of the membrane plane. The RepA-WH1 subunits constituting the pores do not appear to be structurally distorted, because the volume of the model of a RepA-WH1 monomer in its replication-competent conformation fits well within the average electron density (Fig. 7c), whereas some degree of distortion was previously observed in the monomeric building blocks in RepA-WH1 amyloid fibres^35^. This suggests that the possible transformation of ring pores into fibres would be linked to a subsequent transition of RepA-WH1 from a pre-amyloidogenic to an amyloid structure. In addition, we show here that, when assembled in the presence of lipids, RepA-WH1(A31V)-mCherry protofilaments lack lateral association, unlike the mature RepA-WH1 amyloid fibres, most probably due to steric hindrance imposed by the fused fluorescent reporter (Fig. 1 and Supplementary Fig. 1)^35^. Recent studies show that ring-shaped, tubular cytotoxic oligomers of α-synuclein, alike those described here for RepA-WH1, also target membranes and lipid vesicles^43^. Both independent lines of evidence reinforce the significance of the assembly of such oligomeric structures for understanding the general mechanisms of amyloid cytotoxicity.

The mammalian prion PrP, as expressed in *E. coli*, is not a fully infectious agent: it is only after being amplified/replicated *in vitro* in the presence of cofactors when it acquires infectivity, and aPLs, together with nucleic acids, are the most efficient of such co-factors^24^,^33^,^34^,^44^. Similarly, the intrinsically disordered protein α-synuclein experiences conformational changes towards folding and/or aggregation upon binding to nucleic acids^45^ or vesicles including aPLs^46^,^47^. The findings presented here on promotion of RepA-WH1 assembly by aPLs, together with the similar role reported for nucleic acids ligands^22^,^23^,^25^,^48^, highlight that the bacterial prionoid, mammalian PrP and α-synuclein share the same effectors in their pathways towards amyloidogenesis. More specifically, we have shown, both in LUVs (Fig. 2) and GUVs (Fig. 3), that PG and CL are the most efficient PLs in promoting RepA-WH1 aggregation. We could not ascertain the fine structure of these aggregates but, as recently shown for the Aβ(1–40) peptide and liposomes^49^, there may exist an inverse correlation between the order and size of the RepA-WH1 fibres and the dimensions of the lipid vesicles. Interestingly, in *E. coli* aPLs are preferentially located in the inner membrane both at the poles and the division septum, where they contribute to the selective location of specific proteins^50^,^51^,^52^. In this way, PG and CL could guide RepA-WH1 towards the bacterial inner membrane as the initial, triggering stage in amyloid cytotoxicity. Underlining the capacity of the minimal system made of RepA-WH1 and aPLs vesicles to model physiologically relevant events for amyloidosis, CL has been found to be required for mitochondrial membrane leakage by Aβ(1–42), α-synuclein and Tau^53^. Although the focus of the present study was to reconstruct in cytomimetic vesicles a possible route for RepA-WH1 toxicity in bacteria, it would be certainly interesting to explore if this bacterial prionoid would also provide more direct information on the role of membranes in human protein misfolding diseases. In this sense, the inclusion in the vesicles of cholesterol or gangliosides, which have a central role in eukaryotic membrane targeting by amyloids^54^,^55^, should be the subject of future research.

This work also addresses a direct comparison between the amyloidogenesis of RepA-WH1(A31V), as it had been studied so far *in vitro*^22^,^23^,^35^, and its His_6_ and mCherry-tagged form, as studied *in vivo* leading to the characterization of RepA-WH1 as the first synthetic bacterial prionoid^26^,^27^,^29^. Although, according to CD spectroscopy, RepA-WH1 and mCherry behave as essentially independent folding units (Supplementary Fig. 2), our data also indicate that the fusion to mCherry enhances the pro-amyloidogenic potential of RepA-WH1 (Fig. 4). In the full-length RepA, the free energy change linked to the coupling of sequence-specific recognition of DNA at replication origins and protein conformational remodelling is naturally used to enable replication initiation^18^,^19^,^56^,^57^. When fused to mCherry, rather than to the native C-terminal WH2 domain, RepA-WH1 channels such free energy change towards the population of a monomeric and aggregation-prone metastable folding intermediate that leads the pathway towards RepA-WH1 amyloidogenesis^27^.

Most natural functional amyloids found so far in bacteria are secreted extracellularly^15^,^16^. Through secretion, bacteria would protect themselves from amyloid-triggered damage to the inner membrane, as has been documented in this work. Heterologous expression in *E. coli* of proteins involved in human disease usually leads to their aggregation as mono/dipolar inclusion bodies, which exhibit some amyloid character^58^,^59^ but are mildly detrimental to bacterial fitness^60^,^61^. By dynamically assembling diverse aggregated particles with distinct toxicities and amyloidogenicities^27^,^29^, the RepA-WH1 prionoid fulfils in bacteria its role as a proteinopathic agent better than the heterologous expression of Aβ(1–40/42), α-synuclein or β2-microglobulin.

The work presented here constitutes a proof of concept of the feasibility of engineering a minimal, cytomimetic and fully synthetic system to model the targeting and leakage of membranes by disease-related amyloids. Finally, we have shown that this system is amenable to screen for small molecules, such as natural polyphenols, with a broad spectrum against the plethora of amyloidogenic proteins involved in human disease.

## Methods

### Protein expression and purification

H_6_-RepA-WH1(A31V)-mCherry was purified from *E. coli* BL21 cells carrying the pLysS plasmid to lyse cells after freezing–thawing. Bacterial cultures were grown in LB at 30 °C to OD_600 nm_ = 0.45. They were induced with IPTG 0.5 mM and further incubated at 30 °C for 3.5 hours. Protein purification was carried out under native conditions as previously described for RepA-WH1^18^,^22^. Protein concentration was calculated assuming a molar extinction coefficient (610 nm) of 75,000 M^−1^·cm^−1^ per RepA-WH1(A31V)-mCherry chain, and 72,000 M^−1^·cm^−1^ for mCherry^62^. Protein stocks (40 μM) were kept at −70 °C in 0.2 M Na_2_SO_4_, 15 mM Na_2_HPO_4 _pH 6, 5 mM 2-mercaptoethanol, 10% (v/v) glycerol.

### SEC-MALS analysis of H_6_-RepA-WH1(A31V)-mCherry

Static light scattering was used in combination with size exclusion chromatography to separate the dimeric and oligomeric RepA-WH1 fractions. A Superdex 200 10/300 GL column (GE Healthcare) was coupled to a multi-angle light scattering detector (DAWN-EOS, Wyatt) with a refractive index detector (Optilab rEX differential refractometer, Wyatt). The system was equilibrated at 25 °C in 0.1 M Na_2_SO_4_, 0.02 M NaH_2_PO_4_, 1% glycerol at pH 6. Two hundred μL of protein (40 μM) were clarified by centrifugation (13,200 × *g*, 5 min) and then injected into the column and eluted at a flow rate of 0.4 mL·min^−1^. Fractions of 0.5 mL were collected and kept at 4 °C for immediate use. Data were acquired using ASTRA 4.9 (Wyatt), and exported for further analysis in Matlab (Mathworks).

### Purification of *E. coli* internal membrane

Inner membrane (IM) vesicles were isolated from exponential phase cultures of the *E. coli* K-12 strain JM600 essentially as described^63^,^64^. The inner and outer membrane vesicles were separated by sucrose gradient centrifugation^65^, washed and diluted to reach 20 A_280 nm_ units, and stored at −80 °C.

### Phospholipids assay

0.5 mL of a 0.1 M ammonium ferrothiocyanate aqueous solution was added to 1 mL of chloroform, containing the lipids to be determined, and the test tube was vigorously shaken for 60 s. Complex formation between ferrocyanate and phospholipids (Sigma, except *E. coli* phospholipids purchased from Avanti Polar Lipids) brings an aliquot of the water-soluble ammonium ferrothiocyanate, proportional to the lipid concentration, in the chloroform phase. After 30 min incubation to separate the two solvent phases, the chloroform solution was transferred to a quartz cuvette and its A_488 nm_ measured. Phospholipids concentration was determined by comparison with a calibration curve, obtained by using solutions of known titre.

### Preparation of large unilamellar vesicles (LUVs)

Lipids were dissolved at 20 mg·mL^−1^ in chloroform/methanol 2:1 (v/v). Solvent was evaporated with N_2_(g), yielding a lipid film that was vacuum-dried for ≥1 h. Lipid films were hydrated at 37 °C in vesicles buffer (0.020 M NaH_2_PO_4_, 0.1 M Na_2_SO_4_, pH 6). The suspension was subjected to 5 freeze-thaw cycles to generate multilamellar vesicles (MLVs). To prepare LUVs, MLVs were passed 20 times through a 0.1 μm pore size polycarbonate filter (Mini-Extruder, Avanti Polar Lipids). LUVs can be stored at 4 °C for 1 week. For calcein-containing LUVs, 90 mM of this fluorophore was included in the vesicles buffer. Free and LUV-encapsulated calcein were separated by size-exclusion chromatography (Sephadex G-50) in vesicles buffer.

### Preparation of giant unilamellar vesicles (GUVs)

Three alternative methods were used: A) *Hydration in agarose films*^66^. One side of a 24 × 24 mm coverslip was dipped in an aqueous solution of 1% (w/v) agarose (ultra low melting temperature, Sigma-Aldrich), spin-coated at 500–1,000 rpm for 30 s and then dried at 37 °C. Ten μL of lipid solution at 10 mg.mL^−1^ were deposited onto the film by spin-coating at 1,000 rpm for 5 min, and then dried under vacuum for 30 min. Coverslips were mounted in a homemade observation chamber, where the lipid film was hydrated with vesicles buffer plus 0.1 M glucose and incubated for 2 h at 37 °C. When required, proteins were added during the incubation (4 μM final concentration). B) *Droplet transfer*^67^,^68^. Lipids were dissolved in chloroform/methanol 2:1 (v/v), transferred to a glass tube and gently dried using N_2_(g) to produce a thin lipid film, which was dried under vacuum for 1 h. Five ml of mineral oil were then added and mixtures (0.5 mg·mL^−1^ lipids) were incubated for 30 min in a water bath sonifier and stored at 4 °C for up to a week. 500 μL of vesicles buffer plus glucose and 500 μL of the oil lipid mixtures were sequentially added in an Eppendorf tube, resulting in the assembly of an interfacial monolayer of lipids. Fifteen μl of protein solution (4 μM) with 0.1 M sucrose were added to 500 μL of the phospholipid-containing oil and then gently emulsified. This was then poured on top of the oil-lipid mixture, thus resulting in a three-layered sample. Tubes were centrifuged (2,000 rpm, 10 min) to force the emulsion drops to pass through the lipid monolayer. The oil suspension was removed from the top and vesicles washed with 500 μL of vesicles buffer plus glucose and finally sedimented at 2,000 rpm, 10 min. C) GUVs were prepared by *electroformation*^69^ by using a homemade chamber with platinum electrodes. The *E. coli* lipid mixture (5 μl, 10 mg.mL^−1^) was spread on each platinum electrode. After solvent evaporation, chambers were filled with vesicles buffer plus glucose and assembled at 37 °C.

### Measurement of liposome radius by dynamic light scattering

Before measurement, all samples were filtered (0.22 μm). Liposome radii were determined by use of a Protein Solutions photon correlation spectrometer. The incident beam (824 nm) was polarized at a 90° angle with the detector and the scattered signal was averaged over 20 measurements.

### Dye leakage experiments

A) *LUVs*. H_6_-RepA-WH1(A31V)-mCherry was mixed with liposomes (30 μM) in vesicles buffer for 2 h at room temperature. Fluorescence was then monitored in a Varioskan plate reader (Thermo Scientific; 494 nm excitation and 512 nm emission filters, 2 nm bandpass). In kinetic studies, leakage was expressed as the percentage of calcein release at any given time, CR(t)%, and calculated according to Equation 1,


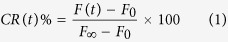


where *F(t)* is the fluorescence intensity of protein-treated LUVs at time t, *F*_*0*_ is the initial fluorescence, and *F*_*∞*_ is fluorescence after complete LUVs disruption by Triton-X (0.2%, v/v). The dependence of calcein leakage on protein concentration was found to fit well to the empirical Hill function (Equation 2),


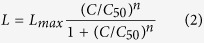


where L is % leakage, C protein concentration (mg·mL^−1^), L_max_ is % leakage at the last time point, C_50_ is the concentration at which C = 1/2 L_max_, and n is a cooperativity parameter. Polyphenolic compounds (DMSO stocks, 0–50 μM final) were tested for inhibition of dye leakage by incubating with protein (0.5 μM) for 10 min in vesicles buffer before addition to LUVs (30 μM). Autofluorescence was measured for each compound in the same buffer in the absence of LUVs and subtracted. Disruption of lipid vesicles by RepA-WH1 in the presence of polyphenols was calculated as % of leakage caused by the protein alone. B) *GUVs*. Calcein (30 μM in 0.1 M sucrose) was included in the phospholipids-oil emulsion as indicated above. Time-elapsed frames were acquired in a confocal laser microscope (see below). For the quantification of inhibition of calcein leakage by polyphenols, 200 μl of vesicles buffer plus glucose and 0.5 μM of protein were placed in an 8-well visualization chamber (LabTech) and incubated for 5 min. Hundred μL of GUVs were added and left to settle for 15 min before imaging.

### Antibody labelling of protein-bound vesicles

200 μL of GUVs prepared using the droplet transfer or electroformation procedures were incubated during 5 minutes with protein, either RepA-WH1(A31V)-mCherry or mCherry. BSA at 2.5% (w/v) was included in all incubations. For immunofluorescence, they were further incubated for 20 min at room temperature with either the monoclonal antibody B3h7 (anti-RepA-WH1 pro-amyloidogenic oligomers^25^; 1:800), or the anti-RepA-WH1 polyclonal antibody α-WH1^16^ (1:400), or an anti-His tag monoclonal antibody (Sigma; 1:200). Samples were then washed 3 times with vesicles buffer plus 0.1 M glucose. Then, GUVs were sequentially incubated for 20 min with an Alexa Fluor-488 conjugated goat anti-mouse antibody (Molecular Probes, Eugene, OR; 1:400). His_6_-tagged proteins were bound to GUVs prepared as described above, but with the addition of 4% Ni^2+^-activated DOGS-NTA (Avanti Polar Lipids).

### Confocal microscopy

GUVs were directly observed at room temperature by confocal microscopy using a Leica TCS-SP5 microscope with a 63X (NA = 1.4–0.60/Oil HCX PL APO) immersion objective. 514 and 488 nm laser lines excited mCherry and calcein, respectively. Precipitated GUVs (400 μl) were placed in an 8-well visualization chamber from LabTech. Quantitative image processing of the green (calcein) channel signal was performed using NIH ImageJ^70^.

### Circular dichroism

CD spectra were recorded in a Jasco-720 spectropolarimeter (Jasco Inc. Easton, MD) over the wavelength range 200–260 nm. Measurements were made in quartz cells with a path length of 0.1 cm in vesicles buffer (0.1 M Na_2_SO_4_, 0.020 M NaH_2_PO_4 _pH 6). Five spectra were averaged for each sample, and the buffer was subtracted as a blank. Raw data were transformed to molar ellipticity [θ] and plotted with Matlab. For thermal denaturation, temperature was increased from 5 °C to 95 °C (at 20 °C/h) monitoring variation in the ellipticity at 220 nm.

### Analytical ultracentrifugation

Hydrodynamic characterization of the proteins, dialysed against vesicles buffer plus 1% glycerol, was achieved by sedimentation velocity at 48,000 rpm with a protein concentration of 0.5 mg·mL^−1^. Experiments were conducted in a Beckman Optima XL-I analytical ultracentrifuge (Beckman Coulter) and sedimentation coefficient distributions were calculated with SEDFIT^71^.

### Assembly of RepA-WH1 fibrils *in vitro*

RepA-WH1(A31V) fibres were built under standard conditions^22^: in a final volume of 100 μL by mixing 20 μM of the purified protein, upon clarification by centrifugation at 13,200 × *g* for 5 min, with dsDNA (*opsp*) or *ex vivo* H_6_-RepA-WH1(A31V)-mCherry aggregates in 40 mM Hepes pH 8, 0.1 M Na_2_SO_4_, 5 mM MgSO_4_, 7% PEG4000, 3% MPD, and leaving the samples at 4 °C for 2–4 weeks. When fibril assembly was explored in the presence of *E. coli* IM vesicles, 5 μL of a preparation of the latter (A_280 nm_≈ 20 units) were mixed with 5 μL of H_6_-RepA-WH1(A31V)-mCherry (40 μM) and 10 μL 0.1 M Na_2_SO_4_, 20 mM NaH_2_PO_4 _pH 6, for 2 h at room temperature. For testing if the assembly of RepA-WH1(A31V) fibres could also be promoted by lipids, 0.004 A_280 nm_ u. of IM were included in the standard incubation (see above) instead of dsDNA or the *ex vivo* aggregate seeds.

### Transmission electron microscopy (TEM)

Protein aggregates were examined on carbon coated 400-mesh copper grids (Ted Pella) after negative staining with 2% aqueous uranyl acetate. Specimen observation was carried out in a JEOL JEM-1230 transmission electron microscope operating at 100 kV and images were captured with a 4k × 4k TVIPS CMOS camera detector (TemCam-F416).

### Single particle EM reconstruction of RepA-WH1 pores

Lipid monolayers were formed at the surface of a 45 μL buffer droplet deposited into a Teflon well by adding 1 μL of *E. coli* lipids (Avanti) at 1 mg·mL^−1^ and incubating at room temperature for 60 min^72^,^73^. An EM grid (see above) was placed on the monolayer, leaving its carbon-coat to interact with the hydrophobic lipid tails. Protein was immediately added at a final concentration of 0.2 μM and incubation was left at room temperature for 15 min. The grid was removed and washed once with water, blotted briefly and stained with 2% uranyl acetate before observation in the experimental setting described above, operated at 100 kV and a final magnification of ×52,000. XMIPP software^74^ was used for general image processing, particle selection, classification and alignment. 20,066 particles were manually selected from 307 micrographs and then aligned and classified using the CL2D algorithm.

## Additional Information

**How to cite this article**: Fernández, C. *et al.* RepA-WH1, the agent of an amyloid proteinopathy in bacteria, builds oligomeric pores through lipid vesicles. *Sci. Rep.* **6**, 23144; doi: 10.1038/srep23144 (2016).

## Supplementary Material

Supplementary Information

Supplementary Movie S1

Supplementary Movie S2

Supplementary Movie S3

## References

[b1] Eichner T. & Radford S. E. A diversity of assembly mechanisms of a generic amyloid fold. Mol. Cell 43, 8–18 (2011). 21726806 10.1016/j.molcel.2011.05.012

[b2] Eisenberg D. & Jucker M. The amyloid state of proteins in human diseases. Cell 148, 1188–1203 (2012). 22424229 10.1016/j.cell.2012.02.022 PMC3353745

[b3] Knowles T. P. J., Vendruscolo M. & Dobson C. M. The amyloid state and its association with protein misfolding diseases. Nat. Rev. Mol. Cell. Biol. 15, 384–396 (2014). 24854788 10.1038/nrm3810

[b4] Hwang D. *et al.* A systems approach to prion disease. Mol. Syst. Biol. 5, 252 (2009). 19308092 10.1038/msb.2009.10 PMC2671916

[b5] Xie H. *et al.* Rapid cell death is preceded by amyloid plaque-mediated oxidative stress. Proc. Natl. Acad. Sci. USA 110, 7904–7909 (2013). 23610434 10.1073/pnas.1217938110 PMC3651444

[b6] David D. C. *et al.* Widespread protein aggregation as an inherent part of aging in *C. elegans*. PLoS Biol. 8, e1000450 (2010). 20711477 10.1371/journal.pbio.1000450 PMC2919420

[b7] Olzscha H. *et al.* Amyloid-like aggregates sequester numerous metastable proteins with essential cellular functions. Cell 144, 67–78 (2011). 21215370 10.1016/j.cell.2010.11.050

[b8] Lashuel H. A. & Butterfield S. M. Amyloidogenic protein-membrane interactions: Mechanistic insight from model systems. Angew. Chem. Int. Ed. 49, 5628–5654 (2010). 10.1002/anie.200906670 20623810

[b9] Milanesi L. *et al.* Direct three-dimensional visualization of membrane disruption by amyloid fibrils. Proc. Natl. Acad. Sci. USA 109, 20455–20460 (2012). 23184970 10.1073/pnas.1206325109 PMC3528594

[b10] Goodchild S. C. *et al.* β2-microglobulin amyloid fibril-induced membrane disruption is enhanced by endosomal lipids and acidic pH. PLoS One 9, e104492 (2014). 25100247 10.1371/journal.pone.0104492 PMC4123989

[b11] Martins I. C. *et al.* Lipids reverts inert Aβ amyloid fibrils to neurotoxic protofibrils that affect learning in mice. EMBO J. 27, 224–233 (2008). 18059472 10.1038/sj.emboj.7601953 PMC2206134

[b12] Campioni S. *et al.* A causative link between the structure of aberrant protein oligomers and their toxicity. Nat. Chem. Biol. 6, 140–147 (2010). 20081829 10.1038/nchembio.283

[b13] Goold R. *et al.* Rapid cell-surface prion protein conversion revealed using a novel cell system. Nat. Commun. 2, 281 (2011). 21505437 10.1038/ncomms1282 PMC3104518

[b14] Tsigelny I. F. *et al.* Role of α-synuclein penetration into the membrane in the mechanism of oligomer pore formation. FEBS J. 279, 1000–1013 (2012). 22251432 10.1111/j.1742-4658.2012.08489.x PMC3925782

[b15] Chapman M. R. *et al.* Role of *Escherichia coli* curli operons in directing amyloid fibre formation. Science 295, 851–855 (2002). 11823641 10.1126/science.1067484 PMC2838482

[b16] Otzen D. & Nielsen P. H. We find them here, we find them there: Functional bacterial amyloid. Cell. Mol. Life Sci. 65, 910–927 (2008). 18034321 10.1007/s00018-007-7404-4 PMC11131872

[b17] Giraldo R., Moreno-Diaz de la Espina S., Fernandez-Tresguerres M. E. & Gasset-Rosa F. RepA-WH1 prionoid: A synthetic amyloid proteinopathy in a minimalist host. Prion 5, 60–64 (2011). 21293179 10.4161/pri.5.2.14913 PMC3166502

[b18] Giraldo R., Andreu J. M. & Díaz-Orejas R. Protein domains and conformational changes in the activation of RepA, a DNA replication initiator. EMBO J. 17, 4511–4526 (1998). 9687517 10.1093/emboj/17.15.4511 PMC1170782

[b19] Giraldo R., Fernández-Tornero C., Evans P. R., Díaz-Orejas R. & Romero A. A conformational switch between transcriptional repression and replication initiation in the RepA dimerization domain. Nat. Struct. Biol. 10, 565–571 (2003). 12766757 10.1038/nsb937

[b20] Fernández-Tresguerres M. E., Martín M., García de Viedma D., Giraldo R. & Díaz-Orejas R. Host growth temperature and a conservative amino acid substitution in the replication protein of pPS10 influence plasmid host range. J Bacteriol. 177, 4377–4384 (1995). 7635822 10.1128/jb.177.15.4377-4384.1995 PMC177187

[b21] Maestro B., Sanz J. M., Díaz-Orejas R. & Fernández-Tresguerres E. Modulation of pPS10 host range by plasmid-encoded RepA initiator protein. J Bacteriol. 185, 1367–1375 (2003). 12562807 10.1128/JB.185.4.1367-1375.2003 PMC142854

[b22] Giraldo R. Defined DNA sequences promote the assembly of a bacterial protein into distinct amyloid nanostructures. Proc. Natl. Acad. Sci. USA 104, 17388–17393 (2007). 17959784 10.1073/pnas.0702006104 PMC2077266

[b23] Gasset-Rosa F., Maté M. J., Dávila-Fajardo C., Bravo J. & Giraldo R. Binding of sulphonated indigo derivatives to RepA-WH1 inhibits DNA-induced protein amyloidogenesis. Nucleic Acids Res. 36, 2249–2256 (2008). 18285361 10.1093/nar/gkn067 PMC2367726

[b24] Silva J. L., Lima L. M., Foguel D. & Cordeiro Y. Intriguing nucleic-acid-binding features of mammalian prion protein. Trends Biochem. Sci. 33, 132–140 (2008). 18243708 10.1016/j.tibs.2007.11.003

[b25] Moreno-del Álamo M., Moreno-Díaz de la Espina S., Fernández-Tresguerres M. E. & Giraldo R. Pre-amyloid oligomers of the proteotoxic RepA-WH1 prionoid assemble at the bacterial nucleoid. Sci. Rep. 5, 14669 (2015). 26423724 10.1038/srep14669 PMC4589793

[b26] Fernández-Tresguerres M. E., Moreno-Díaz de la Espina S., Gasset-Rosa F. & Giraldo R. A DNA-promoted amyloid proteinopathy in *Escherichia coli*. Mol. Microbiol. 77, 1456–1469 (2010). 20662778 10.1111/j.1365-2958.2010.07299.x

[b27] Gasset-Rosa F. *et al.* Direct assessment in bacteria of prionoid propagation and phenotype selection by Hsp70 chaperone. Mol. Microbiol. 91, 1070–1087 (2014). 24417419 10.1111/mmi.12518

[b28] Lindner A. B. & Demarez A. Protein aggregation as a paradigm of aging. Biochim. Biophys. Acta 1790, 980–996 (2009). 19527771 10.1016/j.bbagen.2009.06.005

[b29] Molina-García L. & Giraldo R. Aggregation interplay between variants of the RepA-WH1 prionoid in Escherichia coli. J. Bacteriol. 196, 2536–2542 (2014). 24794561 10.1128/JB.01527-14 PMC4097580

[b30] Soto C. Transmissible proteins: Expanding the prion heresy. Cell 149, 968–977 (2012). 22632966 10.1016/j.cell.2012.05.007 PMC3367461

[b31] Prusiner S. B. Biology and genetics of prions causing neurodegeneration. Annu. Rev. Biochem. 47, 601–623 (2013). 10.1146/annurev-genet-110711-155524 PMC4010318 24274755

[b32] Aguzzi A. Beyond the prion principle. Nature 459, 924–925 (2009). 19536253 10.1038/459924a

[b33] Wang F., Wang X., Yuan C. G. & Ma J. Generating a prion with bacterially expressed recombinant prion protein. Science 327, 1132–1135 (2010). 20110469 10.1126/science.1183748 PMC2893558

[b34] Supattapone S. Synthesis of high titer infectious prions with cofactor molecules. J. Biol. Chem. 289, 19850–19854 (2014). 24860097 10.1074/jbc.R113.511329 PMC4106305

[b35] Torreira E. *et al.* Amyloidogenesis of the bacterial prionoid RepA-WH1 recapitulates dimer to monomer transitions of RepA in DNA replication initiation. Structure 23, 183–189 (2015). 25543255 10.1016/j.str.2014.11.007

[b36] Guerrero-Muñoz M. J., Castillo-Carranza D. L. & Kayed R. Therapeutic approaches against common structural features of toxic oligomers shared by multiple amyloidogenic proteins. Biochem. Pharmacol. 88, 468–478 (2014). 24406245 10.1016/j.bcp.2013.12.023

[b37] Lorenzen N. *et al.* How epigallocatechin gallate can inhibit α-synuclein oligomer toxicity *in vitro*. J. Biol. Chem. 289, 21299–21310 (2014). 24907278 10.1074/jbc.M114.554667 PMC4118093

[b38] Lee J. H. *et al.* Radiating amyloid fibril formation on the surface of lipid membranes through unit-assembly of oligomeric species of α-synuclein. PLoS One 7, e47580 (2012). 23077644 10.1371/journal.pone.0047580 PMC3471876

[b39] Lashuel H. A., Hartley D., Petre B. M., Waltz T. & Lansbury P. T. Jr. Neurodegenerative disease: Amyloid pores from pathogenic mutations. Nature 418, 291 (2002). 12124613 10.1038/418291a

[b40] Lashuel H. A. *et al.* α-Synuclein, especially the Parkinson’s disease-associated mutants, forms pore-like annular and tubular protofibrils. J. Mol. Biol. 322, 1089–1102 (2002). 12367530 10.1016/s0022-2836(02)00735-0

[b41] Ding T. T., Lee S. J., Rochet J. C. & Lansbury P. T. Jr. Annular α-synuclein protofibrils are produced when spherical protofibrils are incubated in solution or bound to brain-derived membranes. Biochemistry 41, 10209–10217 (2002). 12162735 10.1021/bi020139h

[b42] Relini A. *et al.* Monitoring the process of HypF fibrillization and liposome permeabilization by protofibrils. J. Mol. Biol. 338, 943–957 (2004). 15111058 10.1016/j.jmb.2004.03.054

[b43] Chen S. W. *et al.* Structural characterization of toxic oligomers that are kinetically trapped during α-synuclein fibril formation. Proc. Natl. Acad. Sci. USA 112, E1994–E2003 (2015). 25855634 10.1073/pnas.1421204112 PMC4413268

[b44] Srivastava S. & Baskakov I. V. Contrasting effect of two lipid cofactors of prion replication on the conformation of the prion protein. PLoS One 10, e0130283 (2015). 26090881 10.1371/journal.pone.0130283 PMC4474664

[b45] Hegde M. L. & Rao K. S. DNA induces folding in alpha-synuclein: understanding the mechanism using chaperone property of osmolytes. Arch. Biochem. Biophys. 464, 57–69 (2007). 17537399 10.1016/j.abb.2007.03.042

[b46] Kjaer L., Giehm L., Heimburg T. & Otzen D. The influence of vesicle size and composition on alpha-synuclein structure and stability. Biophys. J. 96, 2857–2870 (2009). 19348768 10.1016/j.bpj.2008.12.3940 PMC2711279

[b47] Galvagnion C. *et al.* Lipid vesicles trigger α-synuclein aggregation by stimulating primary nucleation. Nat. Chem. Biol. 11, 229–234 (2015). 25643172 10.1038/nchembio.1750 PMC5019199

[b48] Giraldo R. Amyloid assemblies: Protein *Legos* at a crossroads in bottom-up synthetic biology. ChemBioChem 11, 2347–2357 (2010). 20979077 10.1002/cbic.201000412

[b49] Terakawa M. S., Yagi H., Adachi M., Lee Y. O. & Goto Y. Small liposomes accelerate the fibrillation of amyloid β(1–40). J. Biol. Chem. 290, 815–826 (2015). 25406316 10.1074/jbc.M114.592527 PMC4294504

[b50] Renner L. D. & Weibel D. B. Cardiolipin microdomains localize to negatively curved regions of *Escherichia coli* membranes. Proc. Natl. Acad. Sci. USA 108, 6264–6269 (2011). 21444798 10.1073/pnas.1015757108 PMC3076878

[b51] Oliver P. M. *et al.* Localization of anionic phospholipids in *Escherichia coli* cells. J. Bacteriol. 196, 3386–3398 (2014). 25002539 10.1128/JB.01877-14 PMC4187673

[b52] Laganowsky A. *et al.* Membrane proteins bind lipids selectively to modulate their structure and function. Nature 510, 172–175 (2014). 24899312 10.1038/nature13419 PMC4087533

[b53] Camilleri A. *et al.* Mitochondria membrane permeabilisation by amyloid aggregates and protection by polyphenols. Biochim. Biophys. Acta 1828, 2532–2543 (2013). 23817009 10.1016/j.bbamem.2013.06.026

[b54] Fantini J. & Yahi N. The driving force of alpha-synuclein insertion and amyloid channel formation in the plasma membrane of neural cells: Key role of ganglioside- and cholesterol-binding domains. Adv. Exp. Med. Biol. 991, 15–26 (2013). 23775688 10.1007/978-94-007-6331-9_2

[b55] Grey M. *et al.* Acceleration of α-synuclein aggregation by exosomes. J. Biol. Chem. 290, 2969–2982 (2015). 25425650 10.1074/jbc.M114.585703 PMC4317028

[b56] Díaz-López T. *et al.* Structural changes in RepA, a plasmid replication initiator, upon binding to origin DNA. J. Biol. Chem. 278, 18606–18616 (2003). 12637554 10.1074/jbc.M212024200

[b57] Díaz-López T., Dávila-Fajardo C., Blaesing F., Lillo M. P. & Giraldo R. Early events in the binding of the pPS10 replication protein RepA to single iteron and operator DNA sequences. J. Mol. Biol. 364, 909–920 (2006). 17045290 10.1016/j.jmb.2006.09.013

[b58] Carrió M., González-Montalbán N., Vera A., Villaverde A. & Ventura S. Amyloid-like properties of bacterial inclusion bodies. J. Mol. Biol. 347, 1025–1037 (2005). 15784261 10.1016/j.jmb.2005.02.030

[b59] Wang L., Maji S. K., Sawaya M. R., Eisenberg D. & Riek R. Bacterial inclusion bodies contain amyloid-like structure. PLoS Biol. 6, e195 (2008). 18684013 10.1371/journal.pbio.0060195 PMC2494559

[b60] Lindner A. B., Madden R., Demarez A., Stewart E. J. & Taddei F. Asymmetric segregation of protein aggregates is associated with cellular aging and rejuvenation. Proc. Natl. Acad. Sci. USA 105, 3076–3081 (2008). 18287048 10.1073/pnas.0708931105 PMC2268587

[b61] Winkler J. *et al.* Quantitative and spatio-temporal features of protein aggregation in *Escherichia coli* and consequences on protein quality control and cellular ageing. EMBO J. 29, 910–923 (2010). 20094032 10.1038/emboj.2009.412 PMC2837176

[b62] Shaner N. C. *et al.* Improved monomeric red, orange, and yellow fluorescent proteins derived from *Discosoma sp*. Red fluorescent protein. Nat. Biotechnol. 22, 1567–1572 (2004). 15558047 10.1038/nbt1037

[b63] De Vrije T., Tommassen J. & De Kruijff B. Optimal posttranslational translocation of the precursor PhoE protein across *Escherichia coli* membrane vesicles requires both ATP and protonmotive force. Biochim. Biophys. Acta 900, 63–72 (1987). 3036223 10.1016/0005-2736(87)90278-1

[b64] Jiménez M., Martos A., Vicente M. & Rivas G. Reconstitution and organization of *Escherichia coli* proto-ring elements (FtsZ and FtsA) inside giant unilamellar vesicles obtained form bacterial inner membranes. J. Biol. Chem. 286, 11236–11241 (2011). 21257762 10.1074/jbc.M110.194365 PMC3064179

[b65] Osborn M. J., Gander J. E., Parisi E. & Carson J. Mechanism of assembly of the outer membrane of *Salmonella typhimurium*: Isolation and characterization of cytoplasmic and outer membrane. J. Biol. Chem. 247, 3962–3972 (1972). 4555955

[b66] Horger K. S., Estes D. J., Capone R. & Mayer M. Films of agarose enable rapid formation of giant liposomes in solutions of physiologic ionic strength. J. Amer. Chem. Soc. 131, 1810–1811 (2009). 19154115 10.1021/ja805625u PMC2757642

[b67] Pautot S., Frisken B. J. & Weitz D. A. Production of unilamellar vesicles using an inverted emulsion. Langmuir 19, 2870–2879 (2003).

[b68] Cabré E. J. *et al.* Bacterial division proteins FtsZ and ZipA induce vesicle shrinkage and cell membrane invagination. J. Biol. Chem. 288, 26625–26634 (2013). 23921390 10.1074/jbc.M113.491688 PMC3772209

[b69] Méléard P., Bagatolli L. A. & Pott T. Giant unilamellar vesicle formation from lipid mixtures to native membranes under physiological conditions. Methods Enzymol. 465, 161–176 (2009). 19913167 10.1016/S0076-6879(09)65009-6

[b70] Collins T. J. ImageJ for microscopy. BioTechniques 43, 25–30 (2007). 17936939 10.2144/000112517

[b71] Schuck P. Size-distribution analysis of macromolecules by sedimentation velocity ultracentrifugation and Lamm equation modeling. Biophys. J. 78, 1606–1619 (2000). 10692345 10.1016/S0006-3495(00)76713-0 PMC1300758

[b72] Ford M. G. *et al.* Curvature of clathrin-coated pits driven by epsin. Nature 419, 361–366 (2002). 12353027 10.1038/nature01020

[b73] Szwedziak P., Wang Q., Freund S. M. & Lowe J. FtsA forms actin-like protofilaments. EMBO J. 31, 2249–2260 (2012). 22473211 10.1038/emboj.2012.76 PMC3364754

[b74] Sorzano C. O. *et al.* XMIPP: a new generation of an open-source image processing package for electron microscopy. J. Struct. Biol. 148, 194–204 (2004). 15477099 10.1016/j.jsb.2004.06.006

