# Supplementary material for: RepA-WH1, the agent of an amyloid proteinopathy in bacteria, builds oligomeric pores through lipid vesicles
Source: Sci Rep. 2016 Mar 17;6:23144. doi: 10.1038/srep23144 (PMC4794723; doi:10.1038/srep23144)
Supplement: Supplementary Information [file srep23144-s1.pdf]

SUPPLEMENTARY MATERIALS

RepA-WH1, the agent of an amyloid proteinopathy in bacteria, builds oligomeric pores through lipid vesicles

Cristina Fernández[1], Rafael Núñez[2], Mercedes Jiménez[1], Germán Rivas[1] & Rafael Giraldo[1]

[1] Department of Cellular and Molecular Biology & [2] Electron Microscopy Facility, Centro de Investigaciones Biológicas – CSIC, E28040 Madrid, Spain.

Correspondence should be addressed to R.G (rgiraldo@cib.csic.es)

SUPPLEMENTARY FIGURES

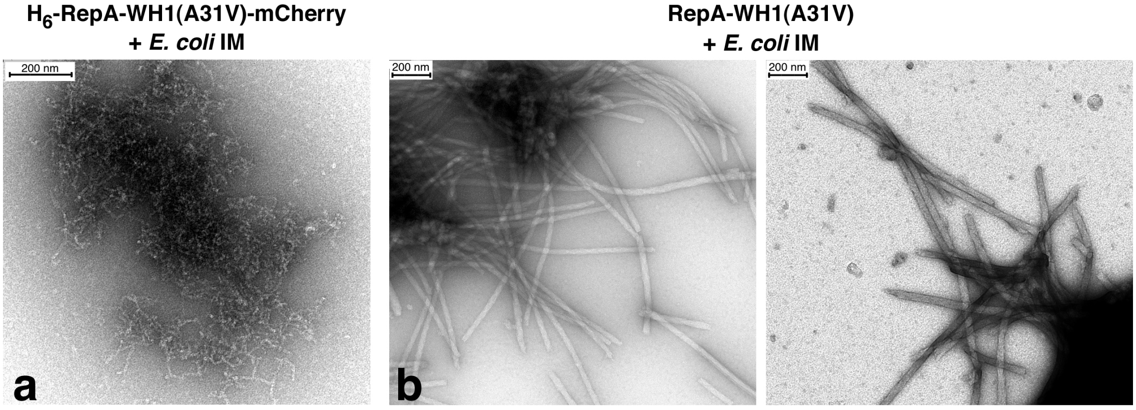

Supplementary Figure 1 Promotion of the assembly of RepA-WH1 amyloid fibres by lipids purified from the *Escherichia coli* inner membrane (IM), as assessed by EM. (**a**) Under standard incubation conditions,[22,35] but including IM as the only effector, the RepA-WH1(A31V)-mCherry chimera assembles as curled single filaments. (**b**) The same assay performed with the RepA-WH1(A31V) protein and IM results in the assembly of the same straight and thick multifilament fibres of the prionoid that are usually observed upon incubation with dsDNA or *ex vivo* RepA-WH1(A31V)-mCherry aggregates.[22,35] The observed difference is compatible with a steric inhibition by the fused mCherry moiety of the lateral association of filaments, which is responsible for building the mature fibres.

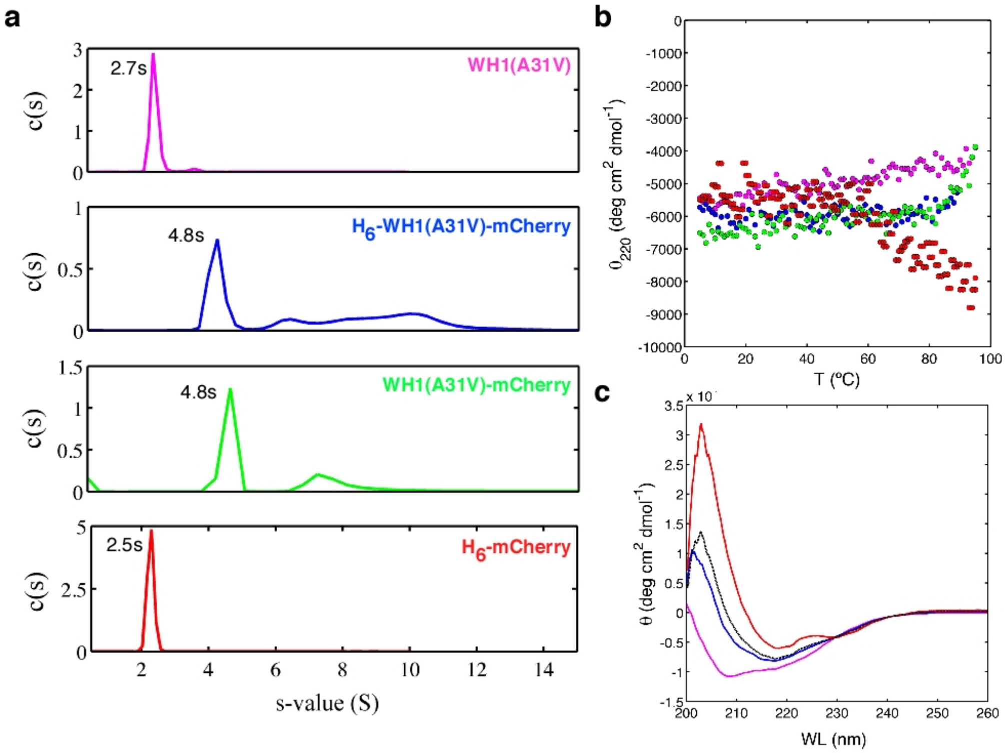

Supplementary Figure 2 Characterization of purified RepA-WH1(A31V)mCherry. (**a**) Sedimentation coefficient (s) distributions of the Lamm equation solution c(s) for H_6-WH1(A31V)-mCherry, WH1(A31V)-mCherry, H_6-mCherry, and WH1(A31V). Data were analysed using a continuous $c(s)$ distribution model as implemented in the program SEDFIT. Up to 30% of H_6-WH1(A31V)-mCherry populates oligomeric states (6-12 S). (**b**) CD thermal denaturation profiles of the same proteins. (**c**) CD spectra at 10 μM concentration of WH1(A31V) (magenta), H_6-WH1(A31V)mCherry (blue) and H_6-mCherry (red) in vesicles buffer at 20°C. The algebraic addition of WH1(A31V) and H_6-mCherry spectra is shown in black.

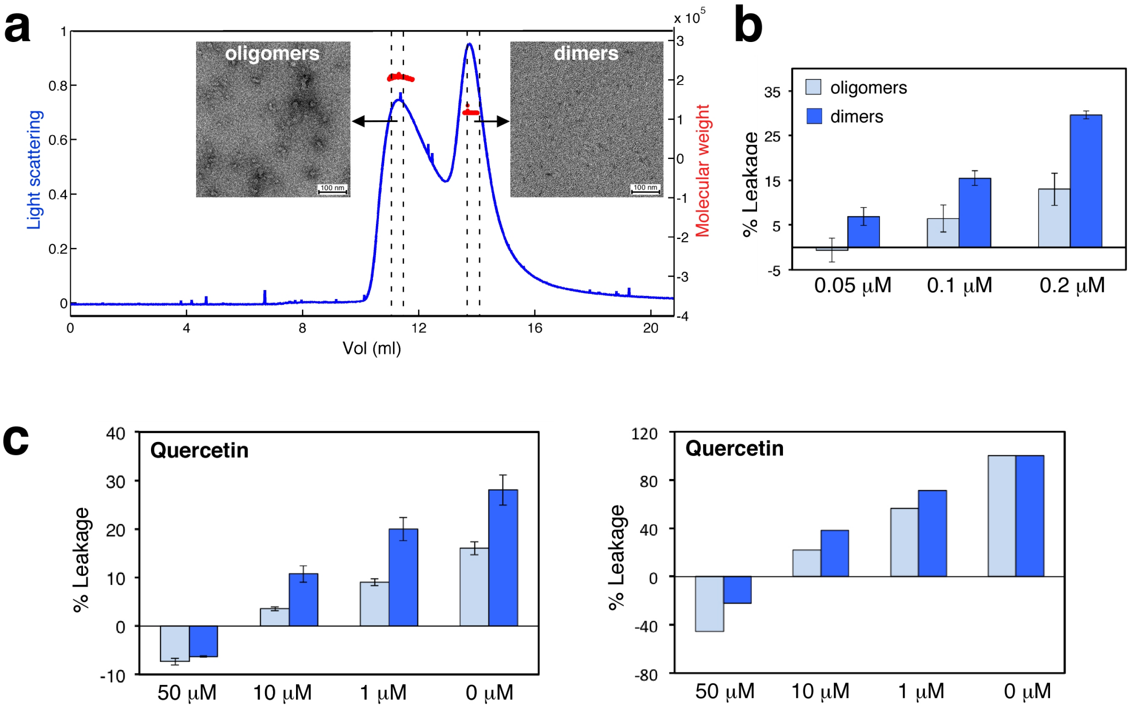

Supplementary Figure 3 Tandem gel filtration and light scattering (SEC-MALS) analysis of purified H_6-RepA-WH1(A31V)-mCherry. (**a**) A representative SEC-MALS profile is shown. The calculated Mw values (right y-axis; in red) and the normalized light scattering intensity at 90° (left y-axis; blue) are plotted. Fractions from oligomers and dimers were visualised by EM. (**b**) Calcein efflux from POPC:POPG (1:1) LUVs (30 μM) incubated with the oligomeric (light blue) and dimeric (dark blue) fractions of the protein at the indicated concentrations. Leakage after complete disruption of all vesicles by Triton X-100 was set to 100%. (**c**) *Left*: Calcein efflux experiments were carried out as in (b) but the protein (0.1 μM) and the flavonoid quercetin (1-50 μM) were incubated for 10 minutes before addition to LUVs. *Right*: Vesicle leakage normalized to the maximum levels achieved by dimers and oligomers in the absence of quercetin, to highlight differences in the protective effect of titration with the flavonoid. All experiments were performed at 25°C.

SUPPLEMENTARY MOVIES

Movie 1. Time-lapsed confocal laser microscopy showing the release of calcein (green) from GUVs as promoted by binding of H_6-RepA-WH1(A31V)-mCherry (red) to the vesicles. An image frame was taken every 4 seconds.

Movie 2. Time-lapsed confocal laser microscopy showing the release of calcein (green) from GUVs as promoted by binding of untagged (His_6 removed) RepA-WH1(A31V)-mCherry (red) to the vesicles. A frame was acquired every 10 s.

Movie 3. Time-lapsed confocal laser microscopy showing the disability of H_6-mCherry (red) to release calcein (green) from GUVs. An image frame was taken every 10 s.
